# Structure and Function Studies of Asian Corn Borer *Ostrinia furnacalis* Pheromone Binding Protein2

**DOI:** 10.1038/s41598-018-35509-x

**Published:** 2018-11-20

**Authors:** Suman Mazumder, Salik R. Dahal, Bharat P. Chaudhary, Smita Mohanty

**Affiliations:** Department of Chemistry, Oklahoma State University, Stillwater, OK 74078 USA

## Abstract

Lepidopteran male moths have an extraordinarily sensitive olfactory system that is capable of detecting and responding to minute amounts of female-secreted pheromones over great distances. Pheromone-binding proteins (PBPs) in male antennae ferry the hydrophobic ligand across the aqueous lymph to the olfactory receptor neuron triggering the response. PBPs bind ligands at physiological pH of the lymph and release them at acidic pH near the receptor while undergoing a conformational change. In *Anthereae polyphemus* PBP1, ligand binding to the hydrophobic pocket and its release is regulated by two biological gates: His70 and His95 at one end of the pocket and C-terminus tail at the other end. Interestingly, in Asian corn borer *Ostrinia furnacalis* PBP2 (OfurPBP2), critical residues for ligand binding and release are substituted in both biological gates. The impact of these substitutions on the ligand binding and release mechanism in OfurPBP2 is not known. We report here overexpression of soluble OfurPBP2 and structural characterization at high and low pH by circular dichroism (CD) and NMR. Ligand binding and ab initio model development were carried out with fluorescence and small-angle X-ray scattering (SAXS) respectively. OfurPBP2 in solution at pH 6.5 is homogeneous, well-folded and has a compact globular shape.

## Introduction

Chemical sensing is extremely important for the survival of most animals. Indeed, chemoreception regulates the most fundamental behaviors in animals, including locating food, mates, and oviposition sites and avoiding enemies. Insects use insoluble fatty acid derivatives that are endowed with “odor/smell” as highly specific signaling molecules for intra and inter-species communications. These volatile smell molecules are also known as semiochemicals. Pheromones are a class of semiochemicals that trigger natural behavioral response in members within the same species. Lepidopteran male moths have an extraordinarily sensitive olfactory system that is capable of sensing airborne pheromone molecules released by females and responding to them over great distances^1^. Pheromone binding proteins (PBPs), present in high concentration in the sensillar lymph of the male moth antennae, play an important role in chemoreception and signaling. These small, highly soluble proteins transport the chemical signal through the aqueous sensillar lymph to the olfactory receptor neuron (ORN), where it is ultimately translated into an electrical signal triggering the male moth response^2,3^.

PBPs comprise a subfamily of OBPs in insects, which were first identified in the giant silk moth *Antheraea polyphemus*^4,5^. These acidic proteins have molecular masses between 14–16 kDa with six conserved cysteine residues^6^. Based on studies carried out on both recombinant PBPs^7–17^ as well as native PBPs isolated from moth antennae^4,15^, it is widely accepted that these carrier proteins pick up the hydrophobic pheromone molecules at pH above 6.0 (pH of sensillar lymph)^7–12^ and transport them across the sensillar lymph releasing at acidic pH near the membrane-bound olfactory receptor by undergoing a dramatic conformational switch^7,8,13–16^. Based on extensive structure-function studies on *Antheraea polyphemus* pheromone-binding protein1 (ApolPBP1), *Bombyx mori* PBP (BmorPBP), *Amyelois transitella* PBP1 (AtraPBP1) and *Lymantria dispar* PBP2 (LdisPBP2), it is clear that the proteins exist in lipid-bound or open or PBP^B^ conformation at neutral pH, and lipid-free or closed or PBP^A^ conformation at acidic pH^6–17^. In the bound or PBP^B^ conformation, the lipid molecule occupies the binding pocket while the unstructured C-terminus is exposed to the solvent^7–12^. However, in the free or PBP^A^ conformation, the unstructured C-terminus switches to a helix and outcompetes the ligand for the hydrophobic pocket at acidic pH^7,8,13–16^. This conformational switch is controlled by two biological gates: two histidine residues (His70 and His95) at one end of the hydrophobic cavity, and the C-terminus tail of the protein at the other end^10,14,18–20^.

**Figure 1:**
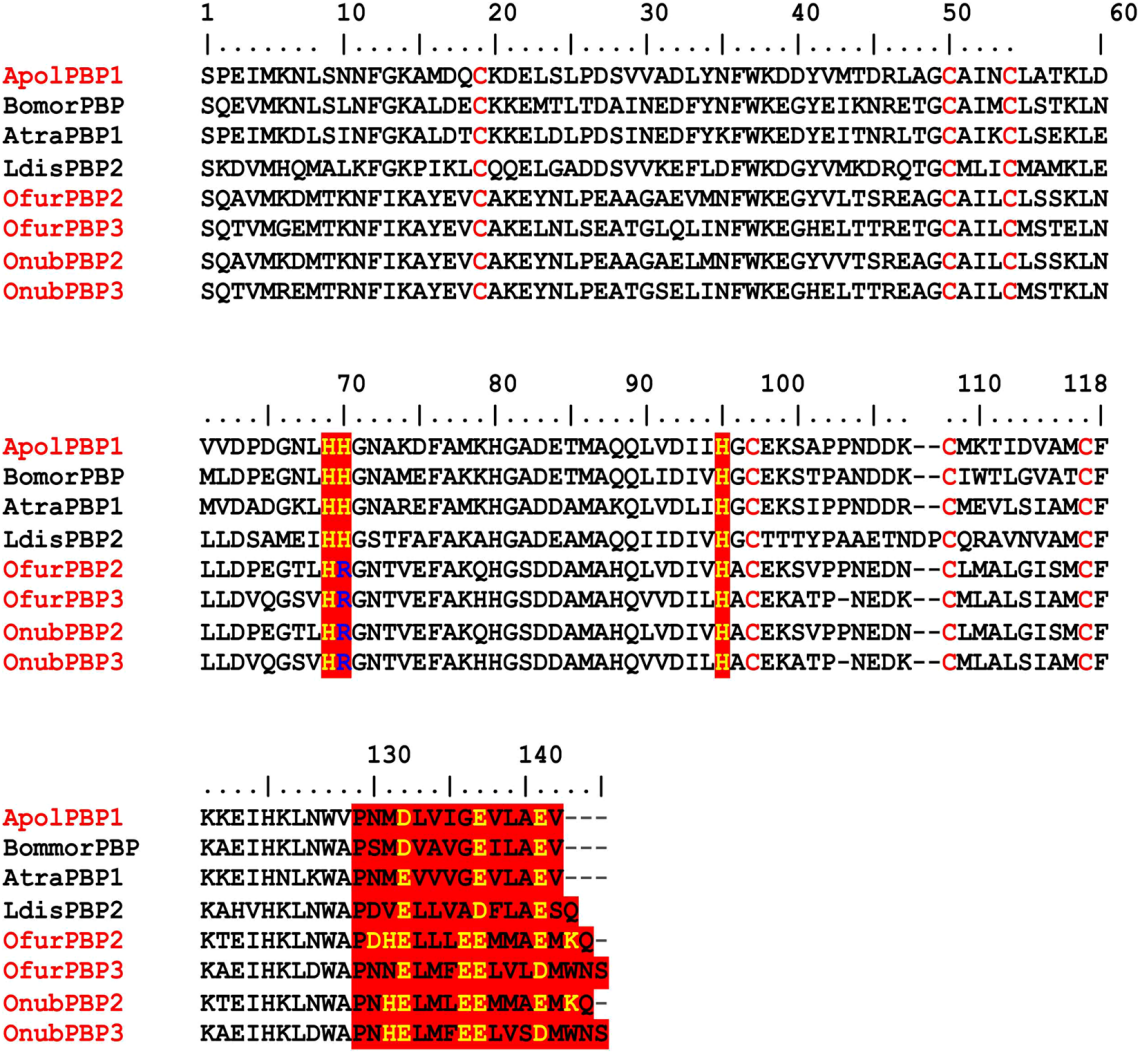
Primary sequences of the PBPs of the moths: *Bombyx mori* (GenBank Accession Number X94987), *Antheraea polyphemus* (Acc. Num. X17559), *Amyelois transitella* (Acc. Num. GQ433364), *Lymantria dispar* (Acc. Num. AF007858), *Ostrinia furnacalis* (Acc. Num. LC027679), *Ostrinia nubilalis* (Acc. Num. AF133643), *Ostrinia furnacalis* (Acc. Num. GU828026), *Ostrinia nubilalis* (Acc. Num. GU828021). Six conserved cysteines are shown on red, histidine gate residues (His70 and His95) are shown in red background highlighting the differences in OfurPBPs and OnubPBPs in blue. C-terminal gate is shown in red background with charged residues being highlighted in yellow.

The genus *Ostrinia*, which belongs to the family Crambidae and order Lepidoptera, includes the Asian corn borer (ACB) *Ostrinia furnacalis*, a voracious pest in Asia, Australia, Africa, the Western Pacific Islands, and parts of United States. It is responsible for the destruction of over one third of the total crops and stored foods^21,22^. Its sister species, the European corn borer (ECB) *Ostrinia nubilalis*, is an agricultural pest over much of the northern hemisphere. These moths cause serious damage to economic crops, accounting for nearly 30% of loss in yield for corn and significant damage to over three hundred other garden crops, such as lima beans, bell peppers, tomatoes, yard-long bean, ginger, pepper, sorghum, millet, okra, cotton, sugarcane etc^22–24^. Both *Ostrinia furnacalis* and *Ostrinia nubilalis* serve as excellent models for the study of pheromone communication. In recent years, efforts made to understand the pheromone communication in *Ostrinia* species have provided much information on various players including stimulators, PBPs, and olfactory receptors^17,25–27^. The male-biased PBPs of both *Ostrinia* species share about 50% sequence identity to their counterparts in other well-studied Lepidopteran moths including *Antheraea polyphemus*, *Bombyx mori, Amyelois transitella*, *and Lymantria dispar* (Fig. 1). They also retain six strictly conserved cysteine residues. However, there are major differences in the two-component biological switch: histidine gate and C-terminal gate. These two gates play a crucial role in pH-driven conformational switch involved in pheromone uptake and release.

**Figure 2:**
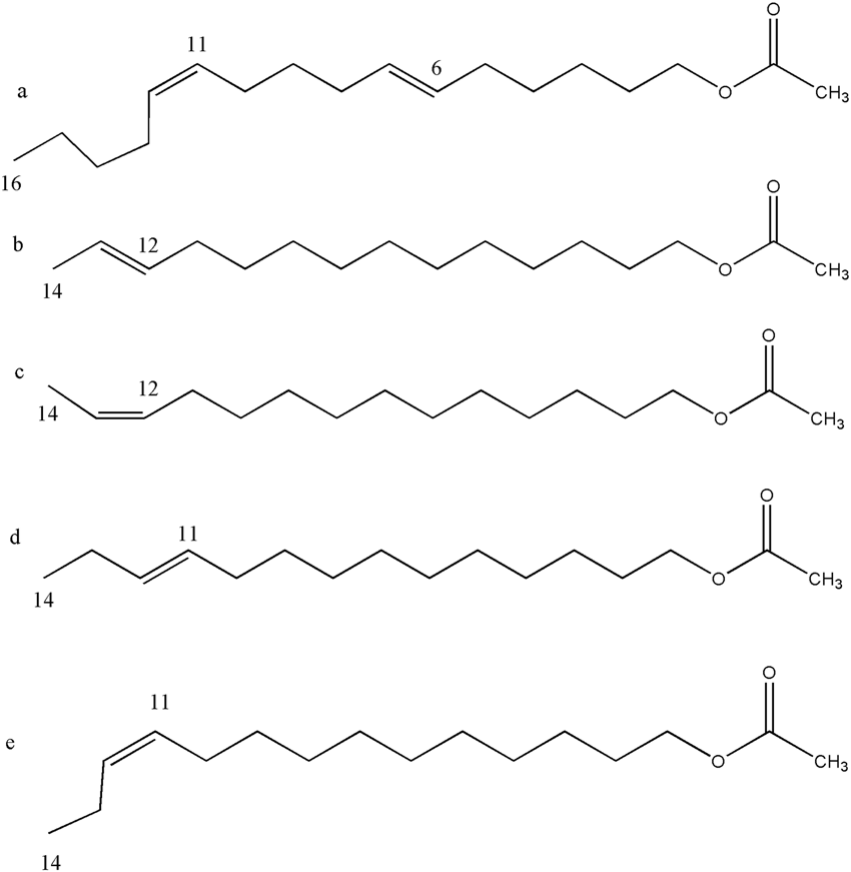
Chemical structures of the pheromone molecules. (**a**) (6E,11Z)-hexadeca-6,11-dienyl-1- acetate of *Antheraea polyphemus*. (**b**) and (**c**) (E) -12 tetradecenyl acetate and (Z)-12-tetradecenyl acetate of *Ostrinia furnacalis*. (**d**) and (**e**) (E)-11 tetradecenyl acetate and (Z)-11-tetradecenyl of *Ostrinia nubilalis*.

OfurPBP2 is a major PBP of male antennae in *Ostrinia furnacalis*^28,29^. Five pheromone-binding proteins from *Ostrinia furnacalis* have been reported^28,29^. Among the five OfurPBPs, PBP2 and PBP3 have been shown to have male-biased expression in the antennae of male species, suggesting that these proteins are involved in the detection of female-secreted pheromone^28^. Females of most *Ostrinia* species use pheromones that are a blend of cis-trans isomers of E-11 and Z-11-tetradecenyl acetate (E11- and Z-11-14:OAc) (Fig. 2). The only exception is females of *O. furnacalis*, which use a blend of E- and Z-12-tetradecenyl acetate (Fig. 2). OfurPBP2 shows 51.4% of sequence identity and 75.7% sequence similarity to ApolPBP1, but differs with respect to the two biological gates: an Arg70 in place of His70 in the His70-His95 gate, and four additional charged residues (Asp130, His131, Glu136 and Lys143) in the C-terminal gate. Both proteins recognize pheromones with acetate functional group but differ in chain length (C-16 for ApolPBP1 and C-14 for OfurPBP2), and the number of double bonds and their positions (Fig. 2). Thus, comparative studies on these two systems will provide insight into pheromone binding and release mechanisms along with pheromone recognition and specificity.

The impact of substitutions of conserved residues in the biological gates on ligand binding and release functions in the OfurPBP2 is not known. In order to address the questions of ligand binding, effect of pH, and the mechanism of ligand release, we have initiated a detailed structural characterization of the Asian corn borer moth *Ostrinia furnacalis* PBP2. We report here an efficient method for the production of pure recombinant soluble OfurPBP2, and structural characterization by circular dichroism (CD), small angle X-ray scattering (SAXS), fluorescence, and high-resolution solution NMR.

## Results

### Cloning, Expression and purification

PBP2 gene of *Ostrinia furnacalis* was cloned into pET21a vector for overexpression of recombinant protein. Recombinant OfurPBP2 expression was optimized using various *E. coli* strains, different temperatures and IPTG concentrations. *E. coli* Origami2 cells were the best hosts for the expression of OfurPBP2 as a soluble protein. OfurPBP2 was purified using a combination of techniques: dialysis, DEAE anion exchange, and finally size-exclusion column chromatography (SEC) using a superdex-75 column fitted to ÄKTA FPLC (GE healthcare). Purity of OfurPBP2 was assessed by SDS PAGE (Supplementary Fig. S1). Pure protein fractions were collected and stored at 4 °C until further use.

### Characterization of OfurPBP2 by MALDI-TOF Mass Spectrometry

**Figure 3:**
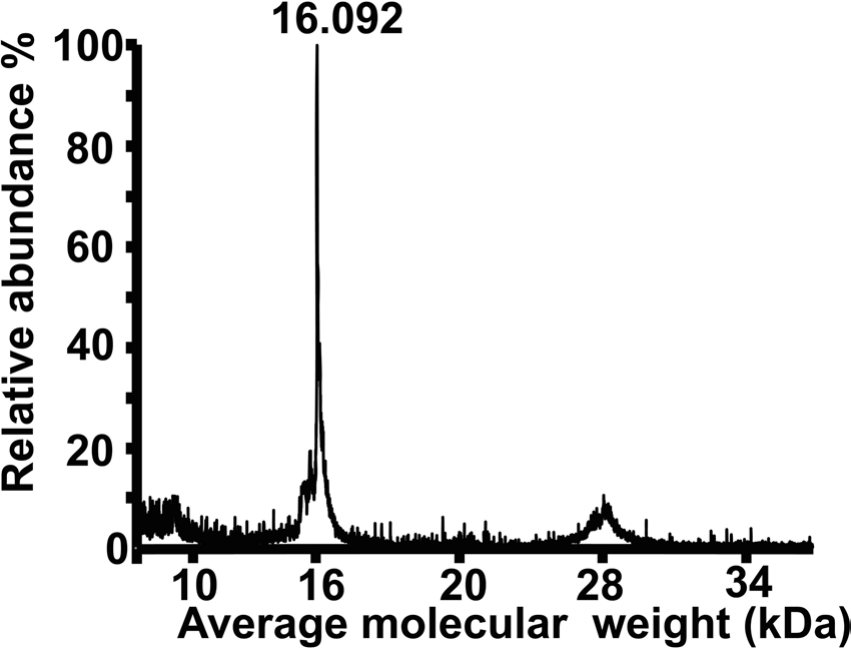
MALDI-TOF analysis of the molecular mass of the purified OfurPBP2. The mass spectrum of the purified OfurPBP2 exhibited a molecular mass of 16.092 kDa, which is in accordance with the calculated value for the OfurPBP2 (16.109 kDa).

An approximate molecular mass can be determined by SDS-PAGE from the relative mobility of a polypeptide chain vs that of a protein marker. However, an accurate mass can be determined by mass spectrometry. The mass spectrum of purified OfurPBP2 exhibited a molecular ion peak at 16.092 kDa, which matched very well to the theoretically calculated molecular mass, 16.109 kDa (Fig. 3).

### Characterization of OfurPBP2 by Fluorescence Spectroscopy

**Figure 4:**
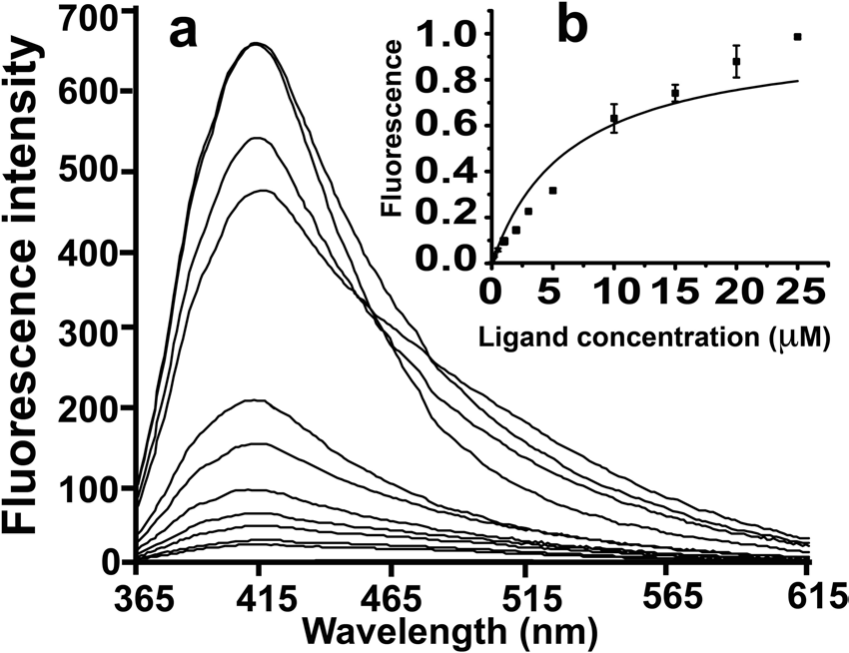
Fluorescence spectra of delipidated OfurPBP2. (**a**) Extrinsic NPN binding fluorescence spectra. The protein concentration was 1 µM in 20 mM phosphate buffer, pH 6.5. Fluorescence emission spectra of OfurPBP2 protein upon addition of different concentration of NPN. The increase in fluorescence intensity is measured at 420 nm. (**b**) Plot of the Relative fluorescence intensity (F_R_) to NPN concentration (in µM), used to calculate the *K*_*d*_ value.

N-phenyl-1-naphthylamine (NPN) is a hydrophobic fluorescent probe, typically used to measure the binding affinity and/or probe the hydrophobic pocket/environment of lipid-binding proteins and membranes. The binding of NPN to delipidated OfurPBP2 at pH 6.5 was measured by monitoring the increase in the NPN fluorescence at 420 nm (Fig. 4a). The change in fluorescence intensity at different ligand concentration was used to calculate the relative fluorescence intensity (F_R_). For determination of dissociation constants, the intensity value corresponding to the maximum fluorescence emission was plotted against the concentration of free NPN, and bound ligand was evaluated from the values for fluorescence intensity. The dissociation constant *K*_*d*_ was determined from a non-linear curve fit of the data (Fig. 4b) using Origin 6.1. The *K*_*d*_ value was calculated to be 6.49 ± 1.01 µM.

### Characterization of OfurPBP2 by far-UV CD Spectroscopy

**Figure 5:**
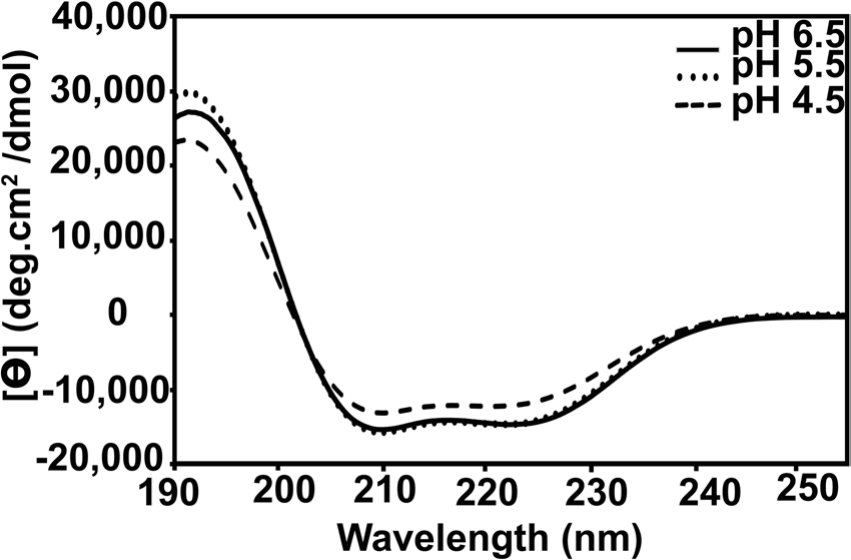
Circular dichroism (CD) spectroscopic analysis of the OfurPBP2 at room temperature. Far UV- CD spectroscopic analysis of OfurPBP2 in 20 mM sodium phosphate buffer at pH 6.5, pH 5.5 and pH 4.5. The protein concentrations were 30 µM. Characteristic minima at 208 and 222 nm at all pH levels are indicative of a highly helical protein.

Far-UV CD spectra of OfurPBP2 at pH 6.5, 5.5 and 4.5 (Fig. 5) had the characteristics of a typical alpha-helical protein with two CD minima: one centering around 208–209 nm and the second around 222–225 nm. However, the CD spectrum at pH 4.5 was quite different from the ones at pH 6.5 and 5.5, suggesting a change in protein structure at this pH. Deconvolution of the CD data with CDPro program suites was performed to calculate the secondary structure content. The deconvolution results indicate the percentage of helix content decreased from 46% at pH 6.5 to 37% at pH 4.5.

### Characterization of OfurPBP2 by NMR Spectroscopy

**Figure 6:**
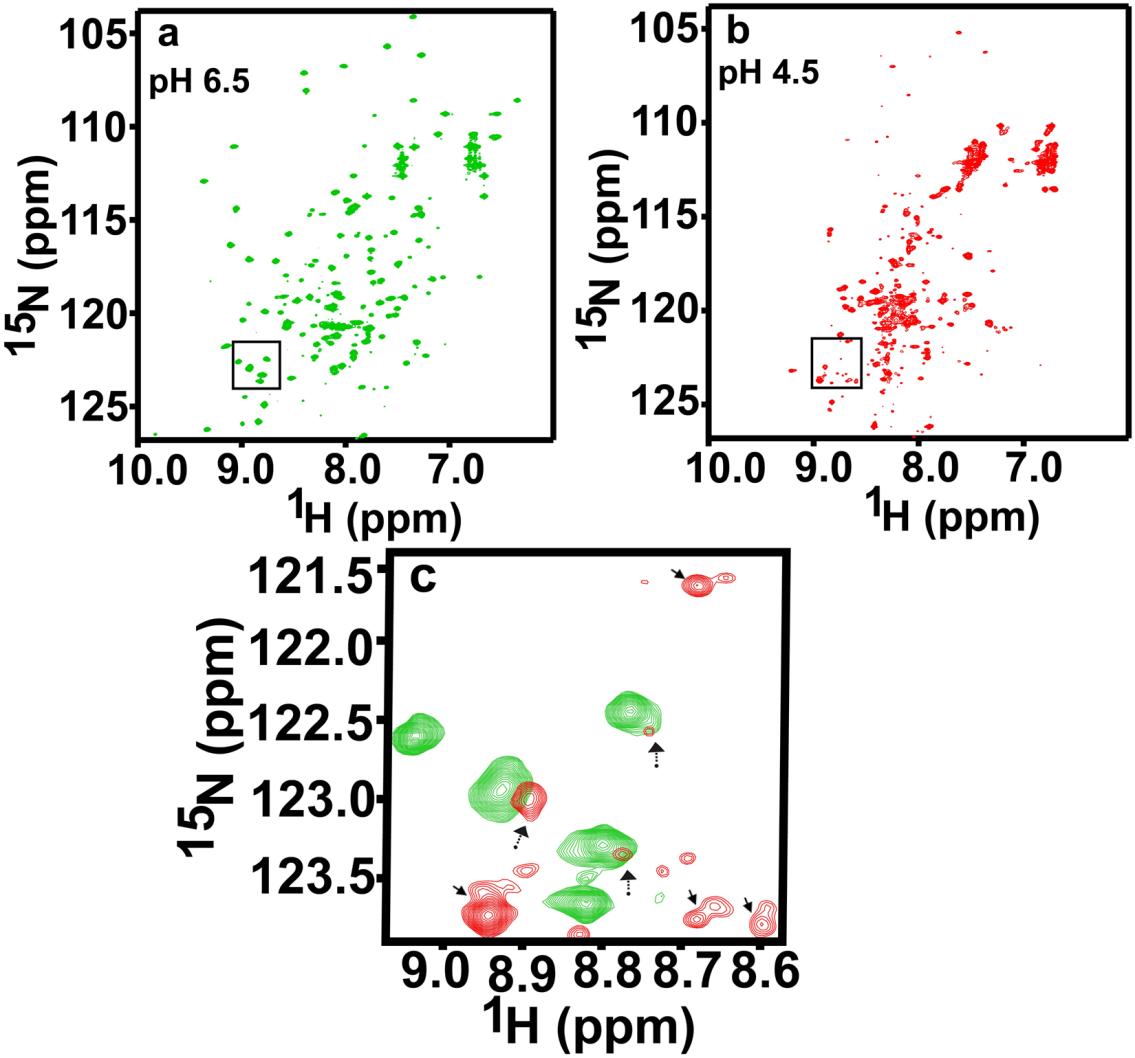
Two-dimensional {^1^H, ^15^N} HSQC spectra of 300 µM OfurPBP2 in 50 mM sodium phosphate buffer, pH 6.5, and 4.5 containing 5% D_2_O, 1 mM EDTA, and 0.01% sodium azide. (**a**) OfurPBP2 at pH 6. 5. (**b**) OfurPBP2 at pH 4.5. (**c**) Overlay region in green (**a**) and red (**b**) are marked by rectangular insets, peak doubling/splitting are indicated by solid arrows and peak intensities reduction are indicated by dotted arrows.

Isotope-labeled pure recombinant OfurPBP2 protein was characterized by NMR. The 2D {^1^H, ^15^N} heteronuclear single quantum coherence (HSQC) spectrum represents the fingerprint of a protein. The quality of resonance dispersion and total number of peaks present in a 2D {^1^H, ^15^N} HSQC NMR spectrum reveals whether a protein is well folded, homogeneous, monomeric heterogeneous or oligomeric. Thus, any change in protein structure or conformation due to mutation/s or ligand binding or changes in pH, temperature or salt concentration etc. are reflected in the HSQC spectrum. The 2D HSQC spectrum of OfurPBP2 shown in Fig. 6a was collected at pH 6.5. The resonances in this spectrum are well-dispersed, indicating that OfurPBP2 is well folded and well behaved at pH 6.5, consistent with the measurements from SAXS.

### Effect of pH on the Conformation of OfurPBP2

To investigate the effect of pH on OfurPBP2 conformation, 2D HSQC NMR experiments were performed at pH 6.5, 6.0, 5.5, 5.0 and 4.5. Spectra determined at pH 6.5, 6.0 and 5.5 showed no significant changes in chemical shift values, suggesting that there was no change in protein conformation (Supplementary Fig. S2). However, the spectral quality began to degrade at pH 5.0 (Supplementary Fig. S3). At pH 4.5, the dispersion of resonances was reduced, with peak crowding at the center of the spectrum (Fig. 6b). On raising the pH back to 6.5, the spectrum returned to its original appearance, demonstrating the reversibility of the change.

### Small Angle X-ray Scattering (SAXS) analysis

**Figure 7:**
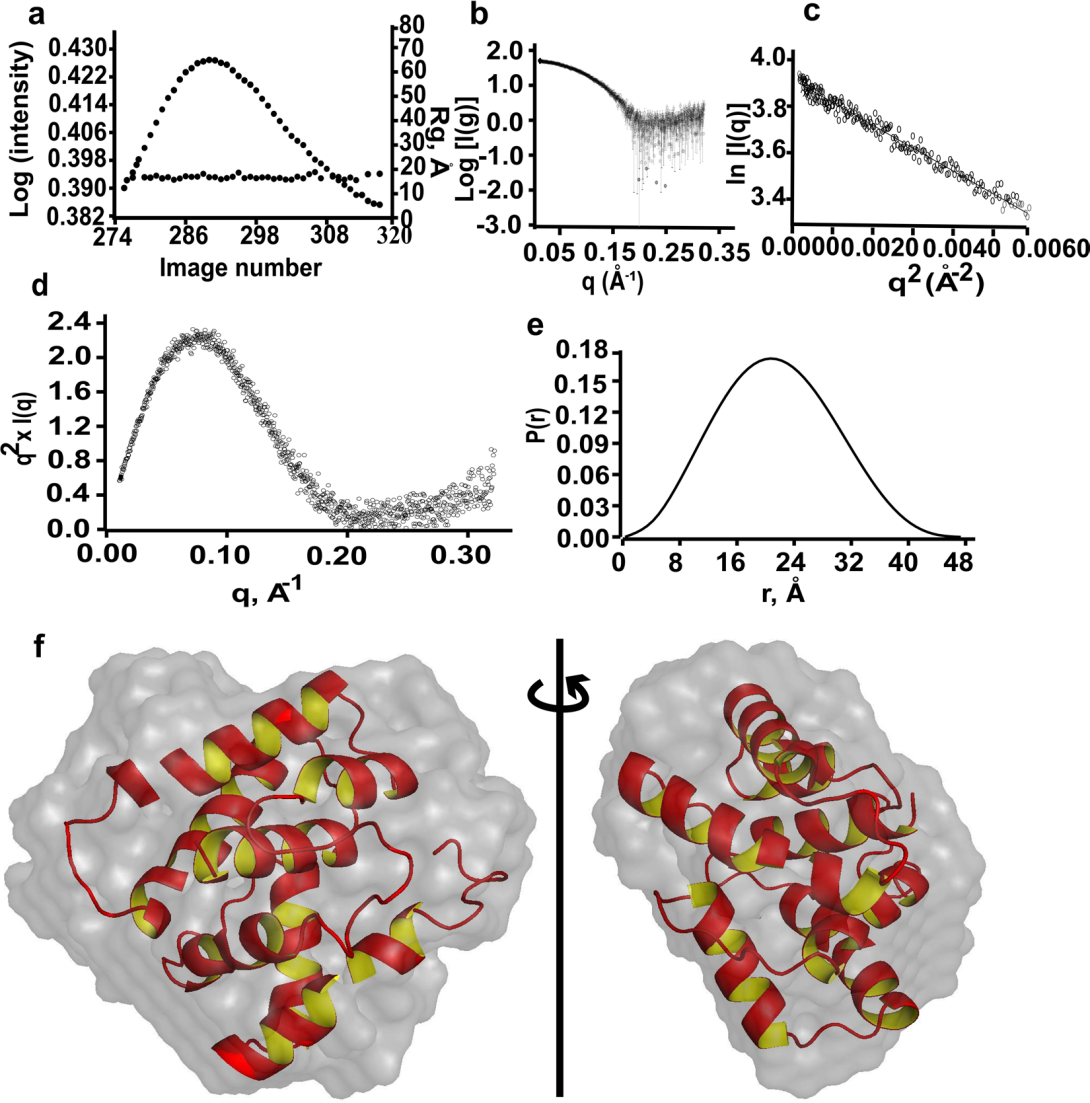
SEC-SAXS data analysis of OfurPBP2. (**a**) SEC-SAXS Signal plot, shown as dotted line across the peak suggests that R_g_ is independent of protein concentration. (**b**) Scattering plot indicates that the protein in solution is homogeneous and monodispersed. (**c**) Guinier plot again confirms the absence of non-specific aggregation. (**d**) Kratky plot suggests that the protein is well-folded and has globular shape. (**e**) Normalized pairwise distribution function shows that OfuPBP2 has globular shape. (**f**) Superposition of SAXS ab initio envelope (grey) with the OfurPBP2 model. The right-hand view is rotated 90 degrees (side view).

Small angle X-ray scattering (SAXS) experiments were performed to develop a model for OfurPBP2. Bio-SAXS is a new structural biology tool that has become popular recently. It is complementary to NMR, cryo-EM, and other techniques. Once purified, homogenous protein sample can be directly characterized by SAXS in solution using various buffer and pH conditions. This is also a non-destructive method similar to NMR. The data provides valuable *in situ* information on protein size, molecular weight, folding/unfolding, protein shape etc. We used SEC-SAXS (size exclusion chromatography small angle x-ray scattering) in HEPES buffer at pH 6.5 so that aggregation or degradation if any during sample shipment is removed. The pure OfurPBP2 was injected to SEC and data were collected as the protein was eluted at each point of the peak (Fig. 7a). The signal plot, shown as a dotted line across the peak, demonstrated that R_g_ of the protein is independent of protein concentration, suggesting that scattering is also independent of concentration (Fig. 7a). The SAXS intensity plots of log[I{q}] vs. q demonstrated that the protein solution was homogeneous and monodisperse without any aggregation or inter-particle interaction (Fig. 7b). The Guinier plot was linear at low q range indicating again monodisperse solution without any non-specific aggregation during data collection (Fig. 7c). The Kratky plot generated a bell-shaped curve with a well-defined maximum which unequivocally established that OfurPBP2 is a homogeneous, well folded, compact globular protein as it follows Porod’s law^30^ (Fig. 7d). The radius of gyration R_g_ was determined from Guinier analysis as 16.96 Å, suggesting a globular protein, which was further confirmed by the symmetrical bell-shaped curve for pairwise distribution function P(r) that indicated that OfurPBP2 has globular shape (Fig. 7e). The *ab initio* molecular models were reconstructed by DAMMIF^31^ and fitted to the predicted model (Fig. 7f). SAXS data collection parameters and scattering parameters are given in Table S1 in the Supplementary Material.

### Homology Modeling

The structure of OfurPBP2 predicted with homology-based modeling (Supplementary Fig. S4) has similarities with the structures of the other lepidopteran PBPs^10–12^. The structure has six α-helices and the C-terminus is unstructured and exposed to the solvent.

### Molecular Docking

**Figure 8:**
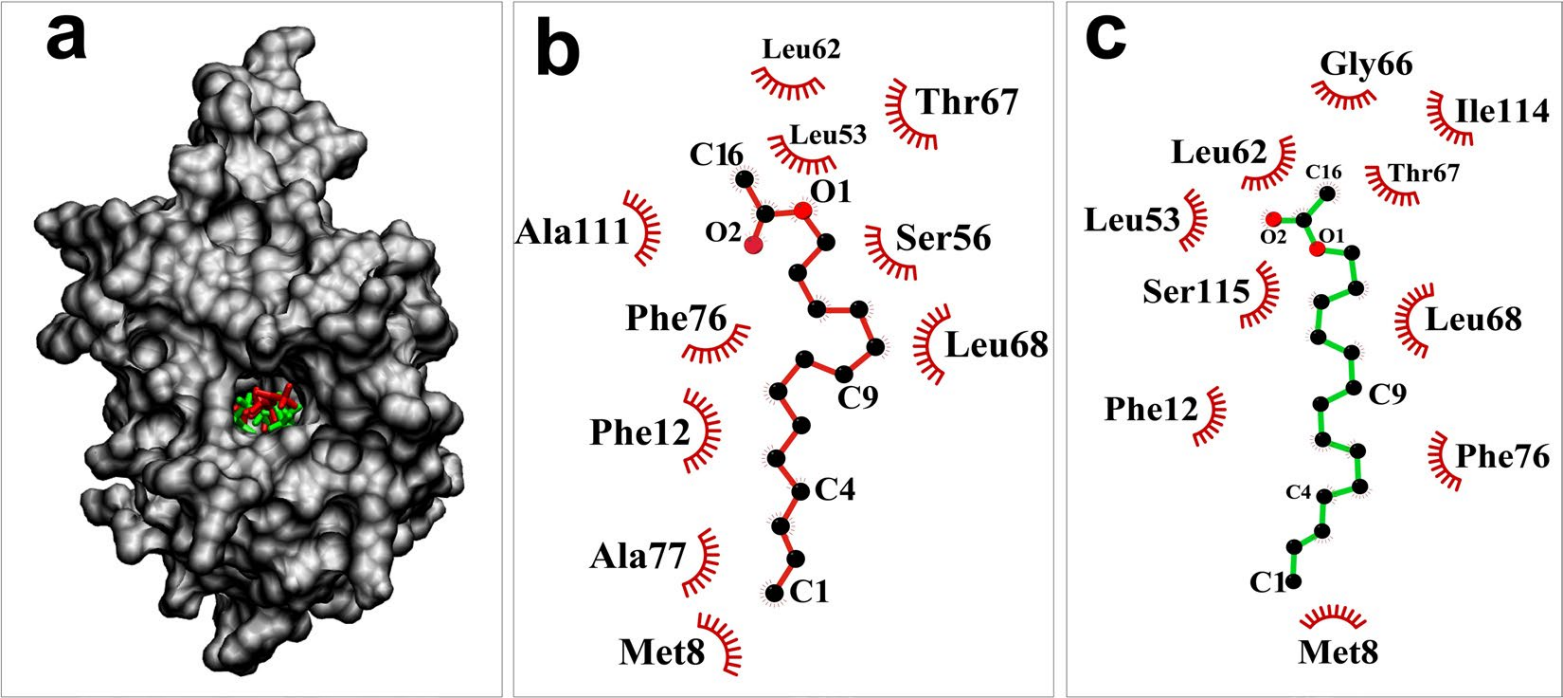
Protein ligand interactions. (**a**) Surface representation of OfurPBP2 protein with E-12(Red) and Z-12(Green)-tetradecaenyl acetate pheromone in the hydrophobic pocket respectively. (**b**) and (**c**) Ligpot showing the hydrophobic interactions of protein with E-12 and Z-12-tetradecaenyl acetate pheromones respectively. Oxygen atom on ligands is represented as solid red circles. The hydrophobic interactions are shown as arcs with spokes radiating towards the ligand atoms they contact.

All E-12 and Z-12 pheromones were docked to the predicted OfurPBP2 model (Supplementary Fig. S4) by using GROMACS. The protein backbone root mean square deviation (RMSD) plots (Supplementary Fig. S5a and b) suggest that the protein-ligand systems are equilibrated and remain stable during 10 ns run. The MD simulations results depicted that the protein has a tunnel-like hydrophobic pocket which is occupied by the ligand (either E12 or Z12 ligand), as shown in Fig. 8a–c.

## Discussion

Understanding the mechanism of pheromone communication at the molecular level is essential if control of an invasive agricultural pest *Ostrinia furnacalis* through sensory inhibition is to be achieved. However, very little structure-function information on the proteins involved in the pheromone signaling pathway of *Ostrinia furnacalis* is known. To our knowledge, there is only one report where five OfurPBPs were expressed in *E. Coli* cells as inclusion bodies (IB) and were denatured and refolded before fluorescence-binding assays were carried out with various ligands^32^. Most importantly, no thorough investigation has been made of the 3D structure and/or the effect of pH on ligand binding and releasing functions of OfurPBP2. Since the primary structure of the OfurPBP2 differs from the ApolPBP1 in the well-studied biological gates, it is of great interest to link the sequence to mode of action. Structural information is necessary to understand the functional importance of the differences in the biological gates of OfurPBP2 and ApolPBP1. The production of soluble OfurPBP2 and its structural characterization with various biophysical techniques conducted here will aid in this endeavor.

The OfurPBP2 gene was amplified, cloned and expression was optimized in Origami 2 cells to produce the soluble recombinant protein. Expression of recombinant OfurPBP2 containing three disulfide bonds as a soluble protein eliminated the need for denaturation, followed by renaturation/refolding. Molecular mass was determined through the mass spectrometry technique to be 16.092 kDa, which is consistent with the theoretical mass of 16.109 kDa (Fig. 3). A binding assay with the fluorescent probe, N-phenyl-1-naphthylamine (1-NPN), was performed to verify that the soluble recombinant protein was well-folded containing a hydrophobic cavity. NPN is an excellent reagent for probing lipocalins. Binding of the dye in the hydrophobic pocket of a lipocalin greatly enhances the quantum yield of the dye. Titration of OfurPBP2 with increasing concentration of NPN resulted in a marked blue shift in emission peak with a corresponding increase in fluorescence intensity, suggesting binding of NPN to OfurPBP2. The dissociation constant for OfurPBP2 binding to NPN was calculated to be 6.50 μM (Fig. 4).

Lepidopteran PBPs are reported to undergo a well-defined dramatic conformational switch when the pH is changed from 6.0 or above to 5.0 or below^7–17^. This pH-driven conformational change has been shown to be associated with ligand binding (at high pH) and release at low pH^6–17^. To investigate the effect of pH on OfurPBP2 conformation at high and low pH levels, we used far-UV CD and high-resolution solution NMR analysis. Far-UV CD spectroscopy is an excellent and non-destructive biophysical tool for probing the secondary structure of a protein in solution. Indeed, this region is quite sensitive to a change in pH, temperature, ligand or mutation/s in the protein. Far-UV CD spectra of recombinant OfurPBP2 at pH levels of 6.5, 5.5 and 4.5 were compared (Fig. 5). At pH 6.5 and 5.5, the secondary structure of OfurPBP2 is similar, suggesting that pH does not affect the protein structure much at these pH levels. However, the CD spectrum recorded at pH 4.5 is quite different compared to pH 6.5 and 5.5. Deconvolution of CD spectra showed approximately a 10% decrease in the percentage of *α*-helix content occurred at pH 4.5, indicating a change in protein structure at acidic pH.

The 2D {^1^H, ^15^N} HSQC is a very sensitive NMR experiment that correlates the amide proton of each residue in a protein to its corresponding nitrogen atom except for proline. This region is considered as the fingerprint of a protein. This region is monitored to investigate the effect of pH or temperature or salts or ligand or mutation/s on a protein conformation. The HSQC spectrum of OfurPBP2 at pH 6.5 is well-dispersed, suggesting that the protein is well-folded with a stable tertiary structure (Fig. 6a). The HSQC spectrum recorded at pH 5.5 matched very well with that at pH 6.5, suggesting no change in the protein conformation. However, the fingerprint region of OfurPBP2 at pH 4.5 is significantly different, with reduction in peak dispersion causing more overlap in the center of the HSQC spectrum (Fig. 6b). There is also decrease in peak intensity along with peak doubling for many resonances, indicating the existence of more than one conformation at pH 4.5 (Fig. 6c). To investigate whether OfurPBP2 is denatured at pH 4.5, the pH level was raised back to 6.5 (and later to 7.5). The HSQC spectrum obtained after raising the pH matched the original spectrum (Fig. 6a) taken at pH 6.5, suggesting that there is no acid-induced denaturation in OfurPBP2 and the conformational heterogeneity at pH 4.5 is reversible. Although reversible, the pH titration data of OfurPBP2 is not similar to what has been observed for ApolPBP1 in our laboratory^7,8,12,14^ or other Lepidopteran PBPs, such as BmorPBP and AtraPBP1^9–11,13,15,16^. In ApolPBP1, the ligand-bound protein is primarily in a PBP^B^ (bound) conformation above pH 6.0 and is a clear mixture of PBP^B^ and PBP^A^ (bound and free) conformations between pH 6.0–5.0, while primarily in a PBP^A^ (free) conformation at pH below 5.0^7^. Thus, at pH 4.5, the PBP^A^ (free) conformation is predominantly present^7^. Similar phenomena have been observed for BmorPBP^9,11,13^, AtraPBP1^16,33^, and LdisPBP2^17^. However, in OfurPBP2 the quality of the HSQC data gradually degrades at pH 5.0 and below with resonance crowding at the center of the spectrum, unlike the PBPs mentioned above. Thus, the pH titration studies by NMR indicates that OfurPBP2 does not behave like other well-studied Lepidopteran PBPs, including ApolPBP1^7,12,14^, BmorPBP^9,11,13^, AtraPBP1^16,33^ and LdisPBPs^17^. Based on this experimental evidence, we speculate that OfurPBP2 may have a different mechanism of pheromone uptake and release. However, a thorough investigation of structure and function is necessary to gain insight into the mechanism of pheromone communication in *Ostrinia furnacalis*.

To further characterize the solution structure and overall shape of OfurPBP2 at high pH, size exclusion chromatography-small angle X-ray scattering (SEC-SAXS) data were collected. Analysis of SAXS intensity data confirmed that OfurPBP2 is monomeric and homogeneous in solution at pH 6.5. (Fig. 7a–c). The radius of gyration (R_g_) obtained from the Guinier approximation is consistent at different concentrations, indicating no aggregation. SAXS data clearly suggest that OfurPBP2 is a monomeric, homogeneous, well-folded, compact globular protein (Fig. 7d,e). Since the atomic-resolution three-dimensional structure of OfurPBP2 has not been determined by either NMR or crystallography, we generated a homology-based model to validate the ab-initio model/shape obtained from solution scattering data. The homology-based model fit very well to the ab initio shape reconstructed based on SAXS data (Fig. 7f). Thus, SAXS data allowed us to obtain a low-resolution, envelope shape model that suggests the molecule in solution behaves as a globular-shaped particle. The theoretical small angle scattering plots of the predicted structure were calculated and compared to the experimental scattering plots. The theoretical R_g_ (16.90 Å) was comparable to the experimental R_g_ (16.94 Å). Similarly, the theoretical maximum particle dimension (Dmax) of the model (47 Å) and the experimental Dmax (49 Å) from SAXS data were very close in values, indicating a good fit and a similar shape.

Computer-aided docking has been extensively employed to determine binding site interactions and form the basis for future structure-function studies^34^. Determination of ligand binding mechanisms is the necessary step to obtain more selective and potent ligands for their potential target. Molecular dynamics (MD) study was conducted to identify the binding site and critical amino acid residues for ligand binding. The MD study shows that the primary interaction between the pheromone and the binding pocket is hydrophobic (Fig. 8a). For the E-ligand, the critical amino acid residues responsible for constitution of the hydrophobic pocket, are Met8, Phe12, Leu53, Leu62, Leu68, Phe76, Ala77, and Ala111 (Fig. 8b). Along with these hydrophobic residues, the ligand-binding pocket contains Ser56 and Thr67 as well. Most likely, these polar amino acids form hydrogen bonds with the polar head group of the ligand. Similarly, for the Z isomer, the critical amino acids are Met8, Phe12, Leu53, Leu62, Gly66, Leu68, Phe76, and Ile114 (Fig. 8c). Along with these hydrophobic residues, the ligand-binding pocket contains Thr67 and Ser115. These two polar amino acid residues likely form H- bonds with the Z isomer. The interactions of E- and Z- pheromones to the predicted 3D structure of OfurPBP3 have been reported through docking studies^32^. Interestingly, in the case of OfurPBP3, all interacting residues were identical for both E- and Z- isomers in contrast to OfurPBP2. In OfurPBP2, the exclusive interactions included: Ala77, Ala111, and Ser56 for E-isomer, and Gly66, Ile114 and Ser115 for Z-isomer. Met8, Phe12, Leu53, Leu62, Thr67, Leu68, and Phe76 of OfurPBP2 are common residues that interact with both E- and Z-isomers. Between OfurPBP2 and OfurPBP3, the common residues in ligand binding interactions are: Met8, Phe12, Leu53, Ser56 (only with E-isomer for OfurPBP2), Phe76 and Ala114 (OfurPBP3). In the case of OfurPBP2, position 114 has Ile, which interacts only with the Z-isomer.

## Conclusion

Because of the economic importance, significant efforts have been made in recent years to understand the pheromone detection system in *Ostrinia* with the envision to develop novel pest control strategies for bio-rational crop protection. We have reported here the over-expression of recombinant OfurPBP2 as a soluble protein and have shown that the protein is monomeric, homogeneous, and highly helical with compact globular shape at pH 6.5. However, at pH 4.5, the protein is less helical and is heterogeneous. Based on the pH titration studies, it is clear that the protein does not switch conformations from bound/open/PBP^B^ at pH 6.5 to free/closed/PBP^A^ conformation at pH 4.5. This behavior is in stark contrast to the current model of pheromone uptake and release by Lepidopteran PBPs^7–17^ that is based on extensive biochemical and structural studies on several moth PBPs including ApolPBP1, BmorPBP, AtraPBP1 and LdisPBP2. It is possible that OfurPBP2 has a new mechanism of pheromone binding and release.

## Methods

### Sub-cloning

To clone full-length OfurPBP2 protein, the open reading frame (ORF) sequence was used as template for the PCR amplification. The gene-specific primers were designed using the high throughput primer design tool. The forward primers: 5′-GGAATTCCATATGTCACAAGCAGTGATGAAAGAC-3′; and reverse: 5′-GCGGATCCTCATTGCTTCATTTCGGCCAT-3′ were used. The PCR amplified fragments were purified and then digested with NdeI and BamHI restriction enzymes, and cloned between the NdeI and BamHI restriction sites of the pET21a vector (Novagen/EMD Millipore). The orientation and sequence of the pET21a/OfurPBP2 was confirmed by DNA sequencing.

### Overexpression of OfurPBP2

The recombinant pET21a/OfurPBP2 plasmid was transformed into *Escherichia coli* Origami 2 cells using the pET21a vector (Novagen/EMD Millipore). Protein expression was optimized using several *E. coli* strains, different temperatures and IPTG concentrations. Saturated LB-ampicillin starter culture was diluted (1:25, v/v) in LB media and grown at 37 °C to an A_600_ of 0.50–0.60. Expression was induced with 1 mM IPTG, and cells were harvested by centrifugation after incubation for 6 hours at 30 °C. For the production of ^15^N labelled protein, cells were grown in M9 minimal media culture containing 0.12% ^15^NH_4_Cl. All other procedures were the same, except that cells were grown for 16 hours after induction with IPTG before harvesting. The cells were harvested by centrifugation at 9000 rpm using a Sorvall LYNX 4000 centrifuge for 20 min at 4 °C and kept frozen at −80 °C until needed.

### Purification of OfurPBP2

The cells were re-suspended in lysis buffer containing Bacterial Protein Extraction Reagent (B-PER) with 1 mM EDTA, 1 mM PMSF and cocktail protease inhibitor and lysed through sonication. The recombinant proteins were purified by a combination of dialysis, anion exchanger DEAE chromatography, and size exclusion chromatography using a Superdex 75 column fitted to an ÄKTA FPLC (GE healthcare). Fractions containing the pure monomeric protein were collected based on SDS PAGE analysis and stored at 4 °C. Protein concentration was calculated from the A_280_ using an extinction coefficient of 15845 M^−1^ cm^−1^ ^35^.

### Delipidation of OfurPBP2 for Fluorescence Binding Assay

The delipidation of OfurPBP2 was performed according to Bette *et al*.^36^. The protein was buffer exchanged to 50 mM sodium citrate buffer at pH 4.5 (buffer A) using a Millipore ultrafiltration concentrator (capacity 15 ml, MWCO 3000). The protein was then incubated overnight with Lipidex^TM^-1000 resin equilibrated with buffer A with shaking at room temperature. The protein was eluted from Lipidex^TM^-1000 with buffer A. The eluted protein was concentrated to 2 ml, incubated again overnight with fresh Lipidex^TM^-1000, and eluted with buffer A. The eluted delipidated OfurPBP2 was concentrated. To prepare fluorescence samples, the delipidated protein was exchanged to 20 mM sodium phosphate buffer pH 6.5.

### Matrix-Assisted Laser Desorption Ionization (MALDI) Time-of-Flight Mass Spectrometry

The protein sample for MALDI-TOF measurement was desalted with a CENTRI-SPIN 10 column (Princeton Separations, NJ). 2,5 Dihydroxybenzoic acid (DHB) was used as the matrix. MALDI mass spectra were recorded on a Voyager DE-PRO MALDI-TOF mass spectrometer (Applied Biosystems).

### Fluorescence Spectroscopy

Fluorescence experiments were performed on a Cary Eclipse Fluorescence Spectrophotometer (Varian) at room temperature with a quartz cuvette (1-cm light pathlength). Both emission and excitation slit widths were set to 10 nm. The sample was excited at 350 nm and emission spectra recorded from 370 to 600 nm. All experiments were repeated at least twice. N-phenyl-1-naphthylamine (NPN) was used as a fluorescent probe. The binding of 1-NPN to delipidated OfurPBP2 at pH 6.5 was measured by monitoring the increase in the NPN fluorescence at 420 nm. All fluorescence measurements were carried out in 20 mM phosphate buffer, pH 6.5 in the presence of 0.3% methanol at 22 °C. Methanol at this concentration had the maximum effect on the NPN fluorescence. Phosphate buffer with appropriate amount of NPN (in methanol) served as control for each data point. To 2 ml of 1 μM delipidated OfurPBP2 solution, small aliquots of 2 mM 1- NPN solution in methanol were added to a final concentration of 0–25 μM. Bound ligand was determined from the values of fluorescence intensity with stoichiometry of 1:1 (ligand: protein) at saturation. To calculate the binding constant, relative fluorescence intensity (*F*_*R*_) of the protein at different NPN concentrations were calculated as (*F*_*c*_ − *F*_*min*_)/(*F*_*max*_ − *F*_*min*_), where *F*_*c*_ is corrected fluorescence intensity at ligand concentration [*C*], *F*_*min*_ is the minimum fluorescence intensity when ligand concentration is 0 µM and *F*_*max*_ is the maximum fluorescence intensity. The data were fitted using Origin 6.1 (OriginLab, Northampton, MA) to a nonlinear curve fit of the plot of (*F*_*c*_ − *F*_*min*_)/(*F*_*max*_ − *F*_*min*_) against [*C*] with the equation corresponding to a single binding site. *K*_*d*_ values were calculated using Equation 1, where, B is the maximum relative fluorescence intensity, y is the relative fluorescence intensity at ligand concentration [x] and K is *K*_*d*_.

1 $$\,y=\frac{B\,\ast \,x}{K+x}$$

### Circular Dichroism (CD)

All circular dichroism (CD) experiments were performed on a Jasco J-810 automatic recording spectropolarimeter using 0.05-cm quartz cell cuvette at room temperature in our laboratory in the Department of Chemistry at Oklahoma State University. The far-UV CD data of OfurPBP2 were collected with a protein concentration of 30 μM in 20 mM phosphate buffer at pH levels of 6.5, 5.5 and 4.5. CD spectra of phosphate buffer at respective pH levels were collected as control. The secondary structure contents were quantified through deconvolution of CD spectra by using CDSSTR, CONTINLL and SELCON3 programs incorporated in CDPro software package^37^.

### Small Angle X-ray Scattering (SAXS)

Small-angle X-ray scattering (SAXS) data were collected at beam line ID-18 at the Advanced Photon Source (APS) at Argonne National Laboratory using SEC-SAXS (size exclusion chromatography small angle x-ray scattering). OfurPBP2 protein was prepared at 20 mg/ml in buffer containing 50 mM HEPES pH 6.5, 0.5 mM EDTA, 0.01% NaN_3._ For in-line SEC–SAXS, 0.5 ml protein samples at 20 mg/ml were loaded onto 24 ml superdex-75 columns (GE Healthcare).

### Data processing and Analysis

Normalization, buffer subtraction, and data reduction to I{q} versus q [(where q = (4π sinθ)/λ), θ is the scattering angle, and λ is the wavelength of radiation, 1.03 Å] were performed with the program ATSAS^38^ and *SCÅTTER*. Radius of gyration (Rg) and zero angle scattering (I(0)) parameters were calculated using both GNOM^39^ and Guinier analysis with the program PRIMUS^40^. Inverse Fourier transform calculations of I{q} to yield pair distribution function, P(r); I(0); Rg, and the maximum dimension (D_max_) were carried out using a q-range of 0.004 to 0.33 Å. Figures were made in PyMOL (DeLano Scientific LLC, San Carlos, CA, USA).

### Structural Model

*Ab initio* shape determination with ATSAS bead modelling program DAMMIF^31^ was performed to generate a three-dimensional structure from one-dimensional scattering data from OfurPBP2. No symmetry restraints were applied; default parameters were used in calculations. Ten independent *ab initio* shapes were reconstructed, averaged, and aligned by using the program DAMAVER^41^. DAMMIN^42^ was used to further refine the averaged shape. The SAXS derived *ab initio* shape OfurPBP2 was superimposed with the homology-based 3D model of OfurPBP2 using PyMOL.

### NMR Measurements

All NMR data were collected at 35 °C either on a Varian 600 MHz NMR spectrometer in the Department of Chemistry at Oklahoma State University or with a Bruker Avance II 800 MHz NMR spectrometer fitted with a triple resonance TCI CryoProbe at National High Magnetic Field Laboratory (NHMFL) at Tallahassee, FL. NMR samples contained ~300 μM uniformly ^15^N-labeled OfurPBP2 in 50 mM phosphate buffer at pH 6.5, 5% D_2_O, 1 mM EDTA and 0.01% (w/v) NaN_3_ in a Shigemi tube. The pH titrations of OfurPBP2 were carried out at pH 6.5, 6.0, 5.5, 5.0, 4.5 and back at pH 6.5. The 2D-{^1^H, ^15^N} heteronuclear single quantum coherence (HSQC) spectra were collected at each pH. All data were processed using NMRPipe^43^ and analyzed by using sparky^44^.

### Molecular Modeling and Docking

The 3-dimensional structure of OfurPBP2 was calculated through homology-based modeling with SWISS MODEL^45–47^ server using the BmorPBP structure at physiological pH (PDB ID: 1LS8)^11^ as the template. Selection of the template was based on a BLAST search and the top-ranked templates and alignments were compared. The template, with highest sequence coverage and sequence identity, was taken to build the model. The total sequence identity between the target protein (OfurPBP2) and the template protein (BmorPBP) was 57%. Thus, to guarantee the quality of the homology model, BmorPBP with the highest level of sequence identity was used as a template to construct the 3D structure of OfurPBP2. From the ensemble of twenty NMR structures, model1 (conformer1) was used as a template because it was the most representative model. Docking studies of the pheromone molecules (E12-tetradecaenyl acetate and Z12-tetradecaenyl acetate) with the protein were performed by carrying out MD simulation using GROMACS v5.1 software package^48^. Coordinates for pheromone molecules were generated using Discovery Studio v17.2.0.1.16349. The pheromone molecules were sketched and edited to provide the correct geometry by using sketch and edit window of the Discovery Studio. The co-ordinates were saved for use in docking studies by GROMACS. Topology files for pheromones were obtained from ATB topology builder^49^. The topology file for protein was prepared using pdb2gmx tool incorporated in GROMACS using Gromos54a7 force field^50^. Protein and ligand were merged for each system, solvated with simple point charge (SPC) water molecules, energy minimized, and equilibrated. Covalent bond lengths were constrained using LINCs algorithm^51^, and the time step was set to 0.002 ps following a published protocol^52^. The molecular dynamics (MD) simulation was carried out for 10 ns. All simulations were performed using Cowboy high-performance computer (HPC) at Oklahoma State University_._ LIGPLOT^53^ program was used to study the protein and ligand interactions.

## Electronic supplementary material

Supplementary Information

## Data Availability

Data Availability statement. Data generated or analyzed during this study are included in this published article (and its Supplementary Information files).
