# Supplementary material for: Structure and Function Studies of Asian Corn Borer Ostrinia furnacalis Pheromone Binding Protein2
Source: Sci Rep. 2018 Nov 20;8:17105. doi: 10.1038/s41598-018-35509-x (PMC6244159; doi:10.1038/s41598-018-35509-x)
Supplement: Supplementary file 1 — Supplementary Information [file 41598_2018_35509_MOESM1_ESM.docx]

**Structure and Function Studies of Asian Corn Borer *Ostrinia furnacalis* Pheromone Binding Protein2**

**Suman Mazumder^#^, Salik R. Dahal^#^, Bharat P. Chaudhary, Smita Mohanty***

**Department of Chemistry, Oklahoma State University**, **Stillwater, OK, USA, 74078**

**^#^ First and second authors contributed equally**

* Corresponding author: Prof. Smita Mohanty, **Department of Chemistry,** Oklahoma **State University, Stillwater, OK, USA, 74078,** Email: [smita.mohanty@okstate.edu](mailto:smita.mohanty@okstate.edu)

**Supplementary Information**

Table S1: SAXS Data Collection Parameters and scattering derived parameters of *Ostrinia furnacalis* pheromone binding protein 2

| Instrument | The BioCat Beamline 18ID (Argonne National Laboratory) |
| --- | --- |
| Wavelength (A °) | 1.03 |
| q range (A°^-1^) | 0.004-0.33 |
| SEC instrument | Superdex75(GE Health Care) |
| Exposure time | Continuous 1 s data-frame measurements of SEC elution |
| Sample concentration (before SEC) | 20 mg/ml |
| Sample to Detector Distance | 1.5 m |
| **Structural Parameters** |  |
| I (0) from guiner anlysis | 47 |
| Rg (A°) | 16.96 |
| q_max_ (A° ^-1^) | 0.286 |
| q Rg (A° ^-1^) | 0.1796-1.2990 |
| **P(r) analysis** |  |
| I (0) (A°) | 50 |
| R(g) (A°) | 16.00 |
| D _max_ (A°) | 47 |
| Chi square (total estimate from GNOM) | 0.83 |
| Porod Volume (A°^-3^) | 26114 |
| MW mass estimated (Porod volume) (Da) | 15361 |
| MW form SAXSMoW (Da) | 15165 |
| MW from BSA as standard (Da) | 14650 |
| MW from Sequence (Da) | 16109 |
| Primary data reduction | SCATTER |
| Data processing | ATSAS |
| Ab initio analysis | DAMMIF |
| Validation and averaging | DAMAVER |
| Three-dimensional graphical representation | PyMol |


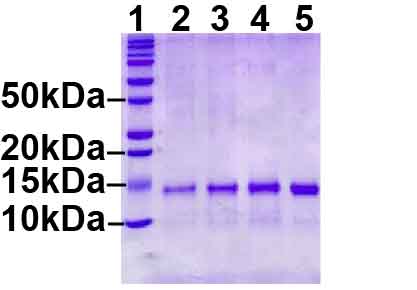
**Fig. S1:**

**Fig. S1**: Coomassie-stained SDS-PAGE of OfurPBP2 purification. The mobility of the protein in SDS-PAGE gel is compatible with its molecular mass. Lane 1: protein molecular weight marker; lanes 2-5: pure protein after size exclusion chromatography. The single protein band after SEC indicates that the protein is very pure.

**Fig. S2:**

**
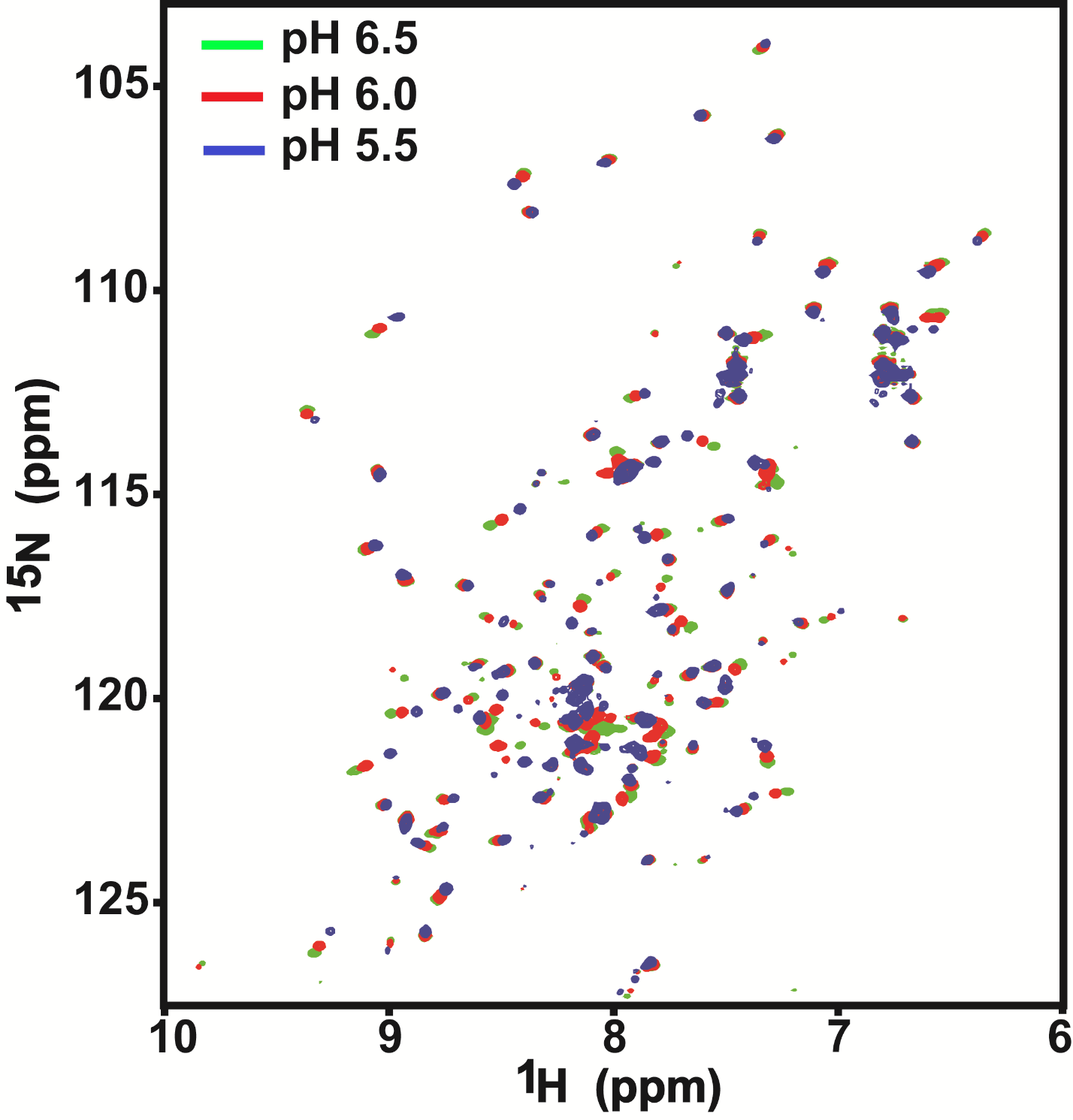
**

Fig S2: Overlay of the 2D {^1^H, ^15^N} HSQC spectra of OfurPBP2 in 50mM sodium phosphate buffer, pH 6.5 (green), 6.0 (red) and 5.5 (blue) containing 5% D_2_O, 1 mM EDTA, and 0.01% sodium azide.

**Fig. S3:**

**
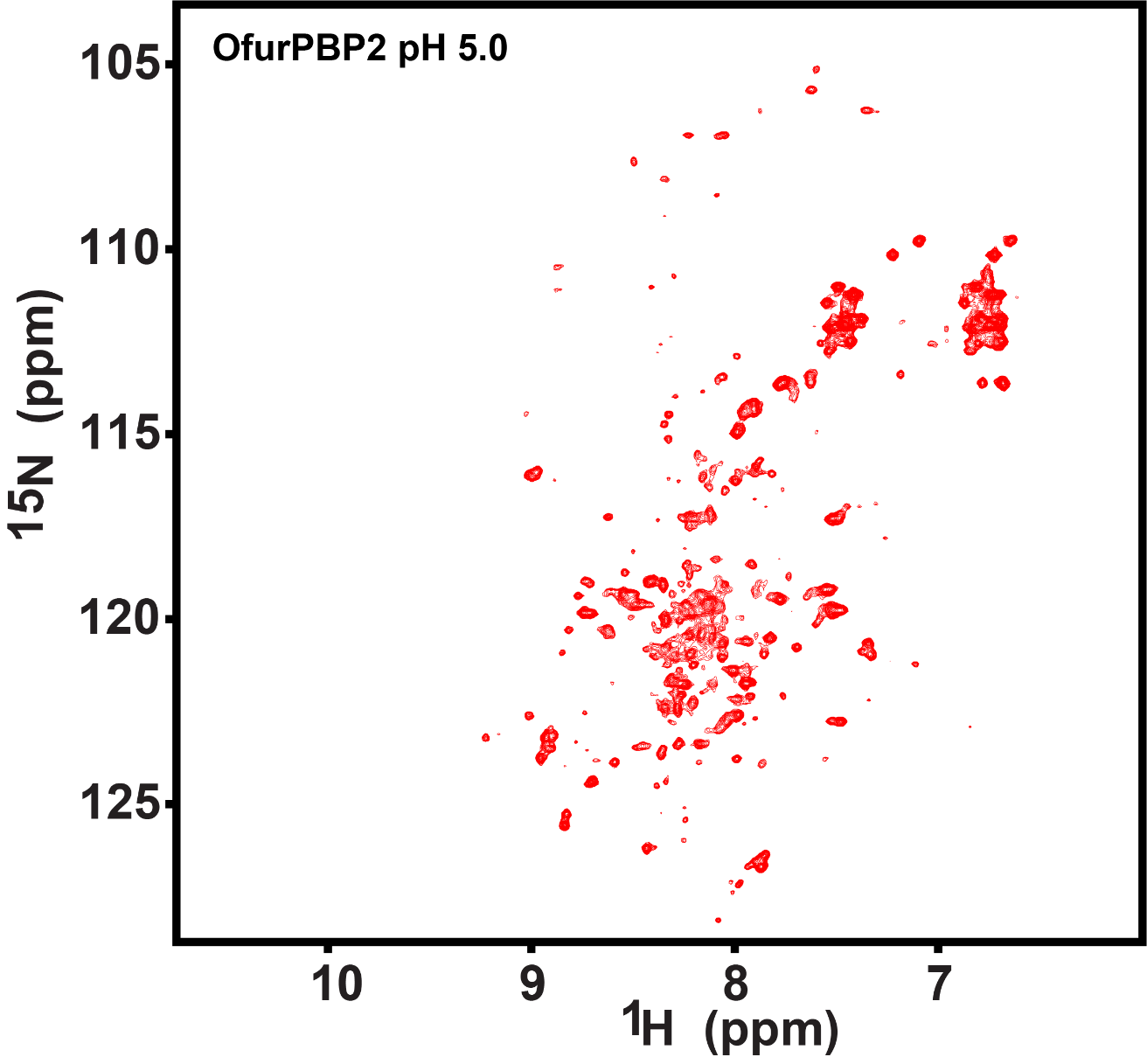
**

Fig S3: 2D {^1^H, ^15^N} HSQC spectrum of OfurPBP2 in 50mM sodium phosphate buffer, pH 5 .0 containing 5% D_2_O, 1 mM EDTA, and 0.01% sodium azide. The spectrum quality started to degrade when the pH is lowered to 5.0.

**Fig. S4:**

**
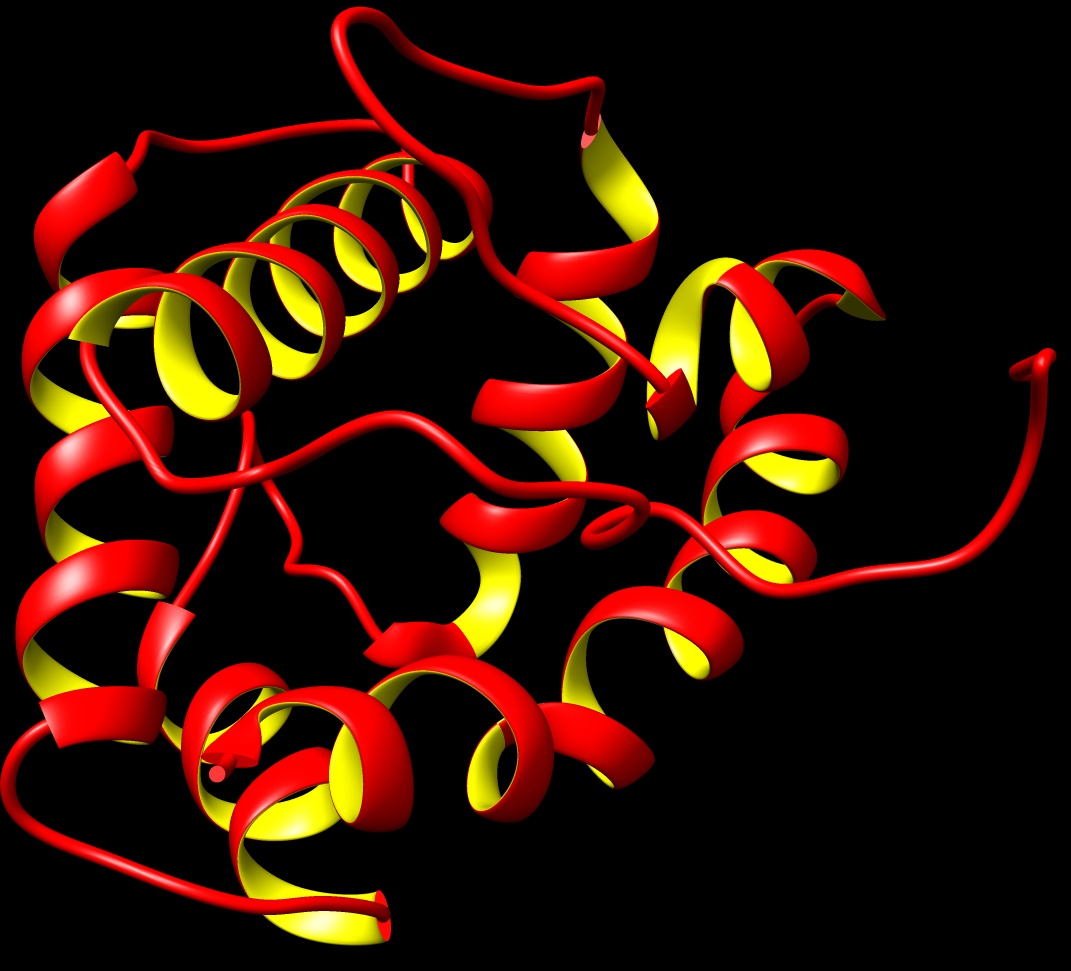
**

Fig S4: Homology based model of OfurPBP2. The predicted structure has many similarities to the other known Lepidopteran PBPs.

**Fig. S5:
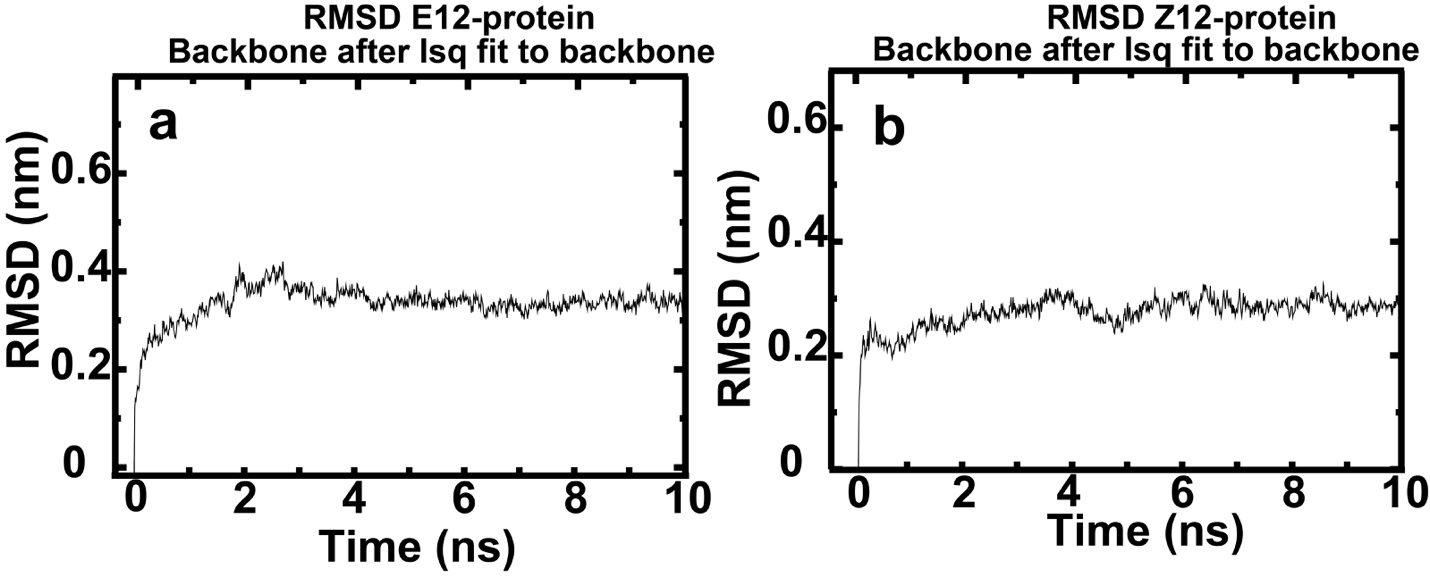
**

Fig. S5 (a) Backbone RMSD of E12-tetradecaeyl acetate pheromone-protein complex. (b) Backbone RMSD of Z12-tetradecaeyl acetate pheromone-protein complex. These plots clearly show that both the systems reached complete equilibrium and the systems were quite stable during 10 ns MD simulations.
